# Jackknife and bootstrapping resampling techniques to evaluate the precision of lens formula constants

**DOI:** 10.1111/aos.17522

**Published:** 2025-05-15

**Authors:** Achim Langenbucher, Jascha Wendelstein, Alan Cayless, Thomas Olsen, Peter Hoffmann, Nóra Szentmáry

**Affiliations:** ^1^ Department of Experimental Ophthalmology Saarland University Homburg Saar Germany; ^2^ Department of Ophthalmology Ludwig‐Maximilian University Munich Munich Germany; ^3^ School of Physical Sciences, The Open University Milton Keynes UK; ^4^ Copenhagen Eye Center and Aros Private Hospital Aarhus Denmark; ^5^ Augen‐ und Laserklinik Castrop‐Rauxel Castrop‐Rauxel Germany; ^6^ Dr. Rolf M. Schwiete Center for Limbal Stem Cell and Aniridia Research Saarland University Homburg Saar Germany; ^7^ Department of Ophthalmology Semmelweis‐University Budapest Hungary

**Keywords:** bootstrap resampling, constant optimisation, formula constant precision, formula constant robustness, jackknife resampling, lens power calculation

## Abstract

**Purpose:**

The purpose of this study was to develop a method for evaluating intraocular lens (IOL) formula constant uncertainties using two modern statistical techniques—jackknife and bootstrap resampling.

**Methods:**

Using two datasets (dataset 1: 888 eyes treated with the aberration correcting Hoya Vivinex IOL, dataset 2: 821 eyes with the spherical Alcon SA60AT/SN60AT IOL), formula constant uncertainties for the SRK/T (Aconst), Hoffer‐Q (pACD), Holladay 1 (SF), simplified Haigis (a0) with preset a1/a2, Haigis (triplet a0/a1/a2), Castrop (triplet C/H/R) and Olsen formula (ACD) were evaluated. All input parameters were jackknife and bootstrap (*N*_*B*_ = 1000) resampled, and formula constants for each sample derived using nonlinear iterative optimisation techniques.

**Results:**

In single constant formulae where the constant acts directly on the effective lens position (Hoffer‐Q, Holladay 1, simplified Haigis, Olsen), the formula constant in each case showed a standard deviation (SD) of about 0.01 with both jackknife and bootstrap sampling. The SRK/T Aconst showed a SD of about 0.018, and the Haigis and Castrop formulae with constant triplets showed large variations in the 3 constants (a0/a1/a2 about 0.036/0.005/0.002, C/H/R about 0.001/0.011/0.012). Direct formula reversion and solving for the formula constant yielded systematically larger SD values (Aconst/pACD,SF/a0/ACD = 0.586/0.395/0.403/0.324/0.304) with highly skewed distributions.

**Conclusion:**

The distributions of formula constants with relevant benchmarks such as SD or confidence intervals can be derived with jackknife and bootstrap resampling techniques, offering potential advantages over direct formula reversion which yields skewed distributions, making central metrics such as the formula constant distribution mean unsuitable for constant optimisation.

## 1 INTRODUCTION

Lens power formulae based on vergence calculations with simplification to the paraxial space are widely used to determine the power of intraocular lenses (IOL) prior to cataract surgery. These formulae are designed to be generally valid for all lens designs and materials (Aristodemou et al., 2011; Langenbucher et al., 2022; Langenbucher, Szentmáry, Cayless, Müller, et al., 2021), biometers and surgical techniques, and are then customised to specific lens types and environmental conditions using formula constants. Optimisation of formula constants requires a representative set of data comprising all preoperative biometric measures used in the formula, the refractive power of the implanted IOL and the refractive outcome in terms of spherical equivalent power after cataract surgery (Langenbucher et al., 2023b). Today, using modern lens formulae with well‐optimised constants, up to 50%/80% of cases in selected study populations result in a refraction within benchmarks of ±0.25/±0.50 dioptres (D) in formula prediction error (Savini et al., 2020; Wendelstein et al., 2022).

The main challenge in formula constant optimisation is that there is no common standard concerning the parameters and metrics to be used. Although the formula prediction error (PE) in terms of spherical equivalent of the achieved refraction minus the spherical equivalent of the formula predicted refraction is undisputedly the most relevant parameter for both the patient and the surgeon, there is still much debate as to the best metric to be zeroed or minimised (Langenbucher et al., 2022; Langenbucher, Szentmáry, Cayless, Müller, et al., 2021). Some surgeons aim to zero the mean or median prediction error, others to minimise the mean or median absolute PE, and others to minimise the root‐mean‐squared PE (Langenbucher, Wendelstein, Szentmáry, et al., 2024). In ophthalmology formulae having a disclosed architecture and a single formula constant, an additional and well‐established option is to reverse the formula and solve for the formula constant (Aristodemou et al., 2011). This results in a ‘perfect’ formula constant for each datapoint in the dataset which maps the preoperative biometry and the power of the implanted lens exactly to the postoperative refraction. However, reverse calculation of the constant is only possible for fully disclosed single constant formulae, and the mean or median of these formula constants from formula reversal does not minimise any of the aforementioned metrics for the PE. In the WEB platform IOLCon (https://iolcon.org), for all formulae, we use an iterative nonlinear optimisation strategy based on minimising the root‐mean‐squared PE. As an alternative, especially for non‐disclosed formulae, some purely data‐driven techniques adapted from artificial intelligence applications such as particle‐swarm optimisation (Langenbucher et al., 2023a) or surrogate model optimisation (Langenbucher, Wendelstein, Cayless, et al., 2024) can be used even when only a black‐box implementation of the formula is available.

Where more than one clinical dataset is available, or with a large dataset that could be split into subsets, the technique of cross‐validation can be used to evaluate the precision or robustness of the generated formula constants. This involves using one dataset or subset for optimisation and subsequently testing the performance against a second dataset or independent subset (Lopez et al., 2023). This enables the standard error or confidence interval of the formula constant to be derived in cases where multiple datasets are available. Alternatively, for fully disclosed single constant formulae such as the SRK/T, Hoffer‐Q, Holladay 1 or the simplified Haigis formula, the standard deviation or the confidence intervals of the formula constants derived using formula reversal on a single dataset could give some insight into formula constant robustness, even though formula reversed constants do not optimise for any metric of PE (Aristodemou et al., 2011; Langenbucher et al., 2022; Langenbucher, Szentmáry, Cayless, Müller, et al., 2021; Schröder et al., 2016; Zhang et al., 2019). However, modern statistical techniques involving resampling provide a means of estimating the standard error or confidence intervals of formula constants using only a single dataset, and these can be used for disclosed and non‐disclosed formulae involving single or multiple formula constants. Two such techniques explored here are *jackknife resampling* and *bootstrapping*. The jackknife works by sequentially eliminating one datapoint from the dataset and then calculating the formula constant, and repeating until each datapoint has been left out once (Zhou et al., 2015; Zuo et al., 2010). It is computationally simpler than bootstrapping and is mostly used to reduce bias and evaluate the variance for an estimator such as our formula constant. Bootstrapping is the most popular resampling technique today and works by sampling with replacement to estimate the distribution of our formula constant. It is mostly used to evaluate the variance of the formula constant distribution (Efron, 1982; Efron & Tibshirani, 1993). Both resampling techniques are very powerful, and they are widely used in statistics to derive relevant metrics from model parameter distributions (Boos & Osborne, 2015; Chavance, 1992; Davison & Hinkley, 1997; DiCiccio & Efron, 1996; Liu et al., 1997; Rodgers, 1999).

The purpose of this paper is

- to present a method for evaluating the precision of formula constants using jackknife and bootstrap resampling techniques,
- to apply this method to two large datasets containing preoperative biometric measures of cataract patients, together with data on the power of the implanted lens and the postoperative refraction, and
- to estimate the standard error and (nonparametric) confidence intervals for the formula constants of the SRK/T, Hoffer‐Q, Holladay 1, Haigis, Castrop and Olsen formulae.

## 2 MATERIALS AND METHODS

### 2.1 Datasets for formula constant optimisation

In this retrospective study, we analysed two clinical datasets from a cataract population from Augen‐ und Laserklinik Castrop‐Rauxel, Castrop‐Rauxel, Germany. These were transferred to us in an anonymised fashion, precluding back‐tracing of the patient. Dataset 1 contains measurements from *N* = 888 eyes (490 right eyes and 398 left eyes; 495 female and 392 male) with the insertion of a 1 piece hydrophobic aspherical (aberration correcting) monofocal intraocular lens (Vivinex XC1 or XY1, Hoya Surgical Optics, Singapore). The mean age was 71.2 ± 9.1 years (median: 71 years, range: 47–91 years). Dataset 2 contains measurements from *N* = 821 eyes (415 right eyes and 407 left eyes; 467 female and 345 male) with the insertion of a 1 piece hydrophobic spherical monofocal intraocular lens (SA60AT or SN60AT, Alcon, Fort Worth, USA). The mean age was 71.7 ± 8.8 years (median: 72 years, range: 49–93 years). The local ethics committee (Ärztekammer des Saarlandes) provided a waiver for this study (157/21). The anonymised data contained complete preoperative biometric records without ‘warning’ or ‘failure’ flags derived with the IOLMaster 700 (Carl‐Zeiss‐Meditec, Jena, Germany) including: axial length AL in mm, anterior chamber depth ACD in mm measured from the corneal front apex to the lens front apex, lens thickness LT in mm, and the corneal front surface radius measured in the flat (R1 in mm) and in the steep meridians (R2 in mm). In addition to the refractive power of the inserted lens (PIOL), the postoperative refraction (spherical equivalent SEQ = sphere +0.5·cylinder) 5–12 weeks after cataract surgery was measured by an experienced optometrist using trial glasses in a trial frame at a refraction lane distance of 6 m and recorded in the dataset. The dataset included only data with a postoperative Snellen decimal visual acuity of 0.8 (20/25 Snellen lines) or higher in order to ensure that the postoperative refraction was reliable. The relevant descriptive data on biometry, PIOL and postoperative refraction are summarised in Table 1. The Excel data (.xlsx‐format) were imported into MATLAB (Matlab 2022b, MathWorks, Natick, USA) for further processing.

**TABLE 1:** Explorative data from preoperative biometry (axial length AL, anterior chamber depth ACD, lens thickness LT, mean corneal radius of curvature R12, mean corneal power K12 derived from corneal curvature using Javal keratometer index, *n*_*K*_ = 1.3375), power of the implanted lens (PIOL) and postoperative refraction (spherical equivalent SEQ) with mean, standard deviation (SD), median, and the lower (CI95lb) and upper (CI95ub) boundary of the 95% confidence interval.

Explorative data		AL in mm	ACD in mm	LT in mm	R12 in mm	K12 in dpt	PIOL in D	SEQ in D
Dataset 1: *N* = 888 Hoya lenses	Mean	24.0980	3.1864	4.6176	7.7646	43.5180	20.6222	−0.5612
	SD	1.4072	0.4081	0.4568	0.2680	1.5006	3.7318	0.9239
	Median	23.9026	3.1848	4.5929	7.7628	43.4763	21.0000	−0.2500
	CI95lb	21.6757	2.3720	3.7333	7.2686	40.6567	12.0000	−2.5000
	CI95ub	27.3514	3.9435	5.5192	8.3012	46.4324	27.5000	0.5000
Dataset 2: *N* = 821 Alcon lenses	Mean	23.1459	3.0440	4.6182	7.6984	43.8927	22.7473	−0.4794
	SD	1.5090	0.4014	0.4336	0.2648	1.5301	4.5718	0.7137
	Median	23.1800	3.0216	4.6100	7.7297	43.6629	22.5000	−0.2500
	CI95lb	20.4510	2.3060	3.8200	77.1135	41.2601	13.5000	−2.6250
	CI95ub	26.4160	3.8180	5.4200	8.1798	47.4451	33.0000	0.5000

### 2.2 Preprocessing of the data

Custom software was written in Matlab. The patient age at the time of surgery was derived from the date of cataract surgery and the date of birth. The corneal radius of curvature R12 was calculated as the harmonic mean R12 = 0.5·R1·R2/(R1 + R2) (Langenbucher, Wendelstein, Szentmáry, et al., 2024), and the mean corneal power K12 was derived from R1 and R2 as Kmean = 0.5·((n_K_ − 1)/R1 + (n_K_ − 1)/R2), where n_K_ is the keratometer index, as indicated in the formula definition. Seven lens power calculation concepts were considered in this paper: the SRK/T formula with formula constant Aconst (Retzlaff et al., 1990; Sanders et al., 1990), Hoffer‐Q formula with formula constant pACD (Hoffer, 1993), Holladay 1 formula with formula constant SF (Holladay et al., 1988), simplified Haigis formula with formula constant a0 and preset constants a1 = 0.4 and a2 = 0.1 (Haigis et al., 2000), Haigis formula with formula constant triplet a0/a1/a2 (Haigis et al., 2000), the Castrop formula with formula constant triplet C/H/R (Langenbucher, Szentmáry, Cayless, Weisensee, et al., 2021; Wendelstein et al., 2022) and the Olsen formula with formula constant ACD (Olsen et al., 1995; Olsen & Hoffmann, 2014). For simplicity and without loss of generality, the corneal thickness was set to 0.55 mm and the corneal back surface curvature was derived from the corneal front surface curvature (R1 and R2) using a preset ratio of front to back surface curvature of 0.84 (Langenbucher, Szentmáry, Cayless, Weisensee, et al., 2021). All formulae included in this analysis were reorganised and solved for the SEQ as a function of preoperative biometrical data and PIOL. The difference between the achieved SEQ (from the postoperative follow‐up examination) and the formula predicted SEQ was taken as the formula prediction error PE (Savini et al., 2020; Schröder et al., 2016).

Formula constants were optimised for both datasets and all formulae using a nonlinear iterative optimisation strategy involving minimising the root‐mean‐squared (RMS) PE of the entire dataset. Formula constant optimisation was implemented using interior point methods (Byrd et al., 2000), which refer to a family of optimisation techniques for solving linear and nonlinear convex optimisations (Boyd & Vandenberghe, 2004; Byrd et al., 1999; Coleman & Li, 1994; Dikin, 1967; Karmarkar, 1984; Waltz et al., 2006). The SEQ prediction was back‐calculated using the optimised constants for each formula, and the prediction error PE was derived.

### 2.3 Jackknife and bootstrap resampling implementation and formula reversal

In the first step, *N* Jackknife samples (with *N* − 1 datapoints each) were derived from each of the datasets by sequentially deleting one datapoint in the dataset (Chavance, 1992; Efron, 1982). For each of the *N* Jackknife samples, the formula constants for all formulae (indicated with a subscript ‘JACK’) were calculated using the nonlinear iterative optimisation strategy as described above. These *N* formula constants were recorded for subsequent analysis. In the second step, *N*_*B*_ = 1000 bootstrap samples were derived from the *N* datapoints using a random resampling strategy with replacement (Davison & Hinkley, 1997; Efron, 1982; Liu et al., 1997). For each of the *N*_*B*_ bootstrapped samples, the formula constants for all formulae (indicated with a subscript ‘BOOT’) were calculated using the nonlinear iterative optimisation strategy as previously described (Boyd & Vandenberghe, 2004; Byrd et al., 1999; Coleman & Li, 1994; Dikin, 1967; Karmarkar, 1984; Waltz et al., 2006). These *N*_*B*_ formula constants were again recorded for subsequent analysis. Finally, all of the single constant formulae (SRK/T, Hoffer‐Q, Holladay 1, simplified Haigis and Olsen formula) were reversed and solved for their respective formula constant, resulting in a ‘perfect’ formula constant for each datapoint in the dataset, mapping the preoperative biometry and the power of the implanted lens exactly to the postoperative SEQ. As before, these *N* formula constants (indicated with a subscript ‘REV’) were recorded for subsequent analysis.

### 2.4 Statistical evaluation

Explorative data are shown in terms of arithmetic mean, standard deviation (SD), median and 95% confidence intervals (CI95, 2.5% quantile as the lower boundary (CI95lb) and 97.5% quantile as the upper boundary (CI95ub)). The mean, SD, median and the CI95 of the respective distributions were recorded in each case for the jackknife and bootstrapped resampled formula constants and for the constants derived from formula reversal. It should be noted that when calculating the SD and the CI95 for the jackknife resampled formula constants, a correction factor of $\sqrt{N-1}$ was applied (Boos & Osborne, 2015; Liu et al., 1997) to account for the loss of degrees of freedom when one datapoint was sequentially deleted from the dataset. In addition, the cumulative distribution function (CDF) was generated for the jackknife resampled formula constants (without correction factor $\sqrt{N-1}$), for the bootstrap resampled formula constants and also for the formula constants calculated from formula reversal. For clarity when showing the distributions for all formulae on a single plot, the CDF for the formula constants have been shifted in terms of zeroing the mean of the distributions, with the corresponding mean values provided in the figure legends.

## 3 RESULTS

Table 1 shows the explorative data of the most relevant input parameters for the datasets 1 and 2 in terms of AL, ACD, LT, R12, K12, PIOL and SEQ. Table 2 relates to dataset 1 and lists the formula constants calculated using the iterative nonlinear optimisation together with the formula constant mean, SD, median and CI95 values for the jackknife samples, the bootstrap samples and the formula reversal. Table 3 relates to dataset 2 and lists the respective formula constants calculated using the iterative nonlinear optimisation together with the formula constant mean, SD, median and CI95 values for the jackknife samples, the bootstrap samples and the formula reversal. From both tables, it can be seen that the mean formula constants from the jackknife resampling match the formula constants derived from the original dataset. The variation in the formula constants from formula reversal is systematically larger as compared to the variations in the formula constants derived from jackknife and bootstrap sampling, respectively.

**TABLE 2:** Formula constants derived from dataset 1 with the result of the iterative nonlinear optimisation, the result of the jackknife resampling, the result of the bootstrap resampling and the result of the formula reversal.

Dataset 1: *N* = 888 Hoya lenses		SRK/T	Hoffer‐Q	Holladay 1	Haigis simplified	Haigis			Castrop			Olsen
		Aconst	pACD	SF	a0	a0	a1	a2	C	H	R	ACD
Nonlinear optimisation		119.2748	5.7356	1.9682	0.3564	−0.6852	0.3417	0.2029	0.3256	0.1477	0.1349	4.7347
*N* = 888 jackknife samples	Mean	119.2748	5.7356	1.9682	0.3564	−0.6852	0.3417	0.2029	0.3256	0.1477	0.1349	4.7347
	SD	0.0182	0.0107	0.0106	0.0097	0.0355	0.0054	0.0017	0.0010	0.0113	0.0123	0.0090
	Median	119.2748	5.7356	1.9681	0.3564	−0.6852	0.3417	0.2029	0.3256	0.1477	0.1349	4.7347
	CI95lb	119.2375	507 157	1.9477	0.3372	−0.7612	0.3303	0.1992	0.3234	0.1232	0.1072	4.7175
	CI95ub	119.3112	5.7582	1.9907	0.3766	−0.6124	0.3531	0.2069	0.3276	0.1727	0.1614	4.7545
*N*_*B*_ = 1000 bootstrap samples	Mean	119.2745	5.7354	1.9689	0.3563	−0.6907	0.3399	0.2033	0.3263	0.1462	0.1323	4.7351
	SD	0.0187	0.0108	0.0110	0.0095	0.0365	0.0053	0.0017	0.0012	0.0108	0.0125	0.0090
	Median	119.2744	5.7350	1.9686	0.3563	−0.6915	0.3397	0.2034	0.3262	0.1467	0.1339	4.7351
	CI95lb	119.2363	5.7140	1.9475	0.3375	−0.7601	0.3296	0.2000	0.3241	0.1236	0.1034	4.7183
	CI95ub	119.3110	5.7577	1.9912	0.3746	−0.6169	0.3508	0.2029	0.3289	0.1659	0.1523	4.7541
*N* = 888 constants from formula reversal	Mean	119.2765	5.8098	2.0143	0.3991							4.7855
	SD	0.5859	0.3946	0.4025	0.3244							0.3037
	Median	119.2854	5.7549	1.9711	0.3682							4.7599
	CI95lb	118.0219	5.1886	1.3540	−0.1664							4.2666
	CI95ub	120.5667	6.7380	2.9459	1.1389							5.4864

*Note*: For the lens formulae with more than one constant formula reversal is not possible. The statistical metrics provided for the jackknife and bootstrap resampling (arithmetic mean, standard deviation SD, median, and the lower (CI95lb) and upper (CI95ub) boundary of the 95% confidence interval) give some insight into the precision or robustness of the formula constants.

**TABLE 3:** Formula constants derived from dataset 2 with the result of the iterative nonlinear optimisation, the result of the jackknife resampling, the result of the bootstrap resampling and the result of the formula reversal.

Dataset 2: *N* = 821 Alcon lenses		SRK/T	Hoffer‐Q	Holladay 1	Haigis simplified	Haigis			Castrop			Olsen
		Aconst	pACD	SF	a0	a0	a1	a2	C	H	R	ACD
Nonlinear optimisation		118.9197	5.4090	1.7001	0.0543	−0.7528	0.2875	0.2019	0.2766	0.1272	0.1365	4.4253
*N* = 821 jackknife samples	Mean	118.9197	5.4090	1.7001	0.0543	−0.7528	0.2875	0.2019	0.2766	0.1272	0.1365	4.4253
	SD	0.0189	0.0109	0.0104	0.0099	0.0370	0.0062	0.0019	0.0043	0.0229	0.0131	0.0105
	Median	118.9186	5.4089	1.7001	0.0542	−0.7528	0.2875	0.2019	0.2766	0.1272	0.1365	4.4253
	CI95lb	118.8834	5.3905	1.6810	0.0373	−0.8212	0.2752	0.1978	0.2683	0.0790	0.1033	4.4084
	CI95ub	118.9554	5.4327	1.7204	0.0743	−0.6708	0.2991	0.2055	0.2866	0.1725	0.1603	4.4528
*N*_*B*_ = 1000 bootstrap samples	Mean	118.9190	5.4085	1.7001	0.0542	−0.7556	0.2870	0.2021	0.2846	0.0929	0.1305	4.4254
	SD	0.0189	0.0110	0.0107	0.0097	0.0364	0.0063	0.0019	0.0027	0.0148	0.0097	0.0105
	Median	118.9186	5.4083	1.7004	0.0545	−0.7554	0.2870	0.2022	0.2836	0.0954	0.1319	4.4251
	CI95lb	118.8834	5.3864	1.6786	0.0347	−0.8265	0.2752	0.1983	0.2816	0.0598	0.1096	4.4054
	CI95ub	118.9554	5.4293	1.7219	0.0730	−0.6856	0.2997	0.2058	0.2912	0.1134	0.1443	4.4463
*N* = 821 constants from formula reversal	Mean	118.8607	5.4791	1.7235	0.0937							4.4812
	SD	0.5657	0.3796	0.3789	0.3179							0.3159
	Median	118.8629	5.4313	1.7019	0.0808							4.4562
	CI95lb	117.7380	4.8983	1.1101	−0.4450							3.9373
	CI95ub	119.9023	6.3214	2.4918	0.8089							5.1507

*Note*: For the lens, formulae with more than one constant formula reversal is not possible. The statistical metrics provided for the jackknife and bootstrap resampling (arithmetic mean, standard deviation SD, median, and the lower (CI95lb) and upper (CI95ub) boundary of the 95% confidence interval) give some insight into the precision or robustness of the formula constants.

**FIGURE 1:**
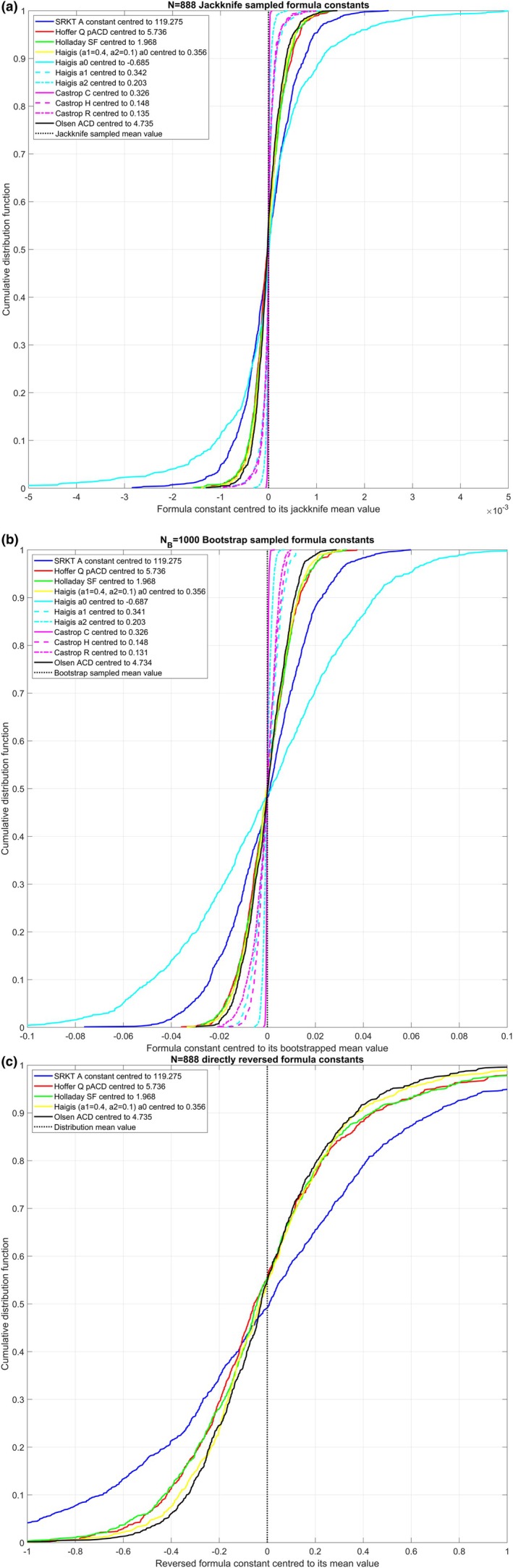
Cumulative distribution plots (CDF) showing the precision or robustness of the formula constants centred to their mean value (indicated in the figure legends) for dataset 1 with *N* = 888 Hoya lenses. The graphs include the Aconst for the SRK/T formula, the pACD for the Hoffer‐Q formula, the SF for the Holladay 1 formula, the a0 for the simplified Haigis formula, the a0/a1/a2 triplet for the Haigis formula, the C/H/R triplet for the Castrop formula and the ACD for the Olsen formula. All formula constants were retrieved using an iterative nonlinear optimisation technique to minimise for the root‐mean‐squared formula prediction error. Subfigure a shows the CDF for the *N* = 888 jackknife resampled formula constants; subfigure b shows the CDF for the *N*_*B*_ = 1000 bootstrap resampled formula constants; and subfigure c displays the CDF for the formula constants derived from the formula reversion (only for single constant formulae). The distributions in subfigure b are broader compared with subfigure b as a consequence of the resampling techniques, and the distributions in subfigure c is much broader compared with subfigures a and b and also somewhat asymmetric with respect to their distribution mean.

Figure 1 relates to dataset 1 with *N* = 888 Hoya lenses and displays the CDF graphs for the formula constant distributions derived from jackknife resampling (subfigure a), bootstrap resampling (subfigure b) and formula reversal (subfigure c). In general, the variation in the lens constants is systematically lower for jackknife resampling (subfigure a) than for bootstrap resampling (subfigure b). From subfigures a and b, it can be seen that the CDF values for those single constant formulae having a constant that directly shifts the mean effective lens position (Hoffer‐Q, Holladay 1, simplified Haigis and Olsen) are very similar, whereas the SRK/T formula (which internally downscales the Aconst with a factor 0.62467 for the conversion to the effective lens position) accordingly shows a larger variation. For the Haigis formula, the variation in the a2 constant is lowest followed by the variation in the a1 constant. This is a consequence of the internal formula architecture in which the effective lens position is derived from a linear superposition of an intercept a0 and a1/a2 upscaled by ACD/AL. Subfigure c, which displays the CDF for the formula constant derived from a direct formula reversal, shows that the formula constant distributions are very broad and asymmetric with respect to the mean, which is indicated by the vertical dashed black line.

**FIGURE 2:**
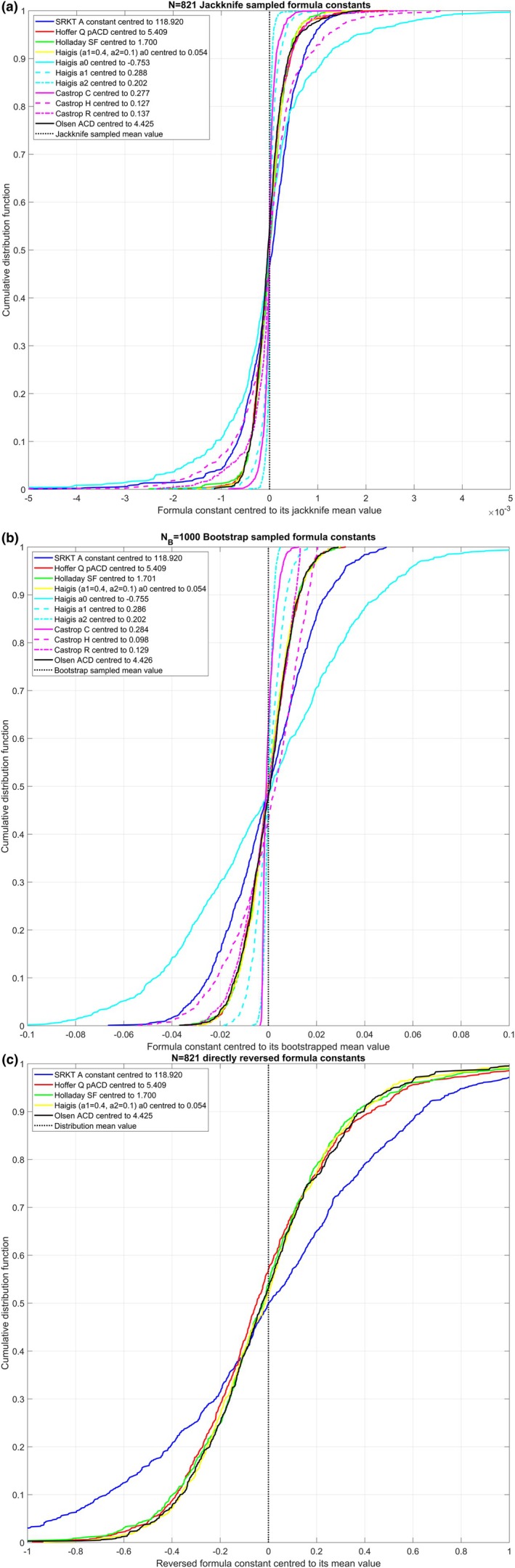
Cumulative distribution plots (CDF) showing the precision or robustness of the formula constants centred to their mean value (indicated in the figure legends) for dataset 2 with *N* = 821 Alcon lenses. The graphs include the Aconst for the SRK/T formula, the pACD for the Hoffer‐Q formula, the SF for the Holladay 1 formula, the a0 for the simplified Haigis formula, the a0/a1/a2 triplet for the Haigis formula, the C/H/R triplet for the Castrop formula and the ACD for the Olsen formula. All formula constants were retrieved using a iterative nonlinear optimisation technique to minimise for the root‐mean‐squared formula prediction error. Subfigure a shows the CDF for the *N* = 888 jackknife resampled formula constants; subfigure b shows the CDF for the *N*_*B*_ = 1000 bootstrap resampled formula constants; and subfigure c displays the CDF for the formula constants derived from the formula reversion (only for single constant formulae). The distributions in subfigure b are broader compared with subfigure b as a consequence of the resampling techniques, and the distributions in subfigure c much broader compared with subfigures a and b and also somewhat asymmetric with respect to their distribution mean.

Figure 2 relates to dataset 2 with *N* = 821 Alcon lenses and displays the CDF graphs for the formula constant distributions derived from jackknife resampling (subfigure a), bootstrap resampling (subfigure b) and formula reversal (subfigure c). Again, the variation in the lens constants is systematically lower for jackknife resampling (subfigure a) than for bootstrap resampling (subfigure b). And we again see very similar CDF values for those single constant formulae having a constant directly that shifts the mean effective lens position (Hoffer‐Q, Holladay 1, simplified Haigis and Olsen), whereas the SRK/T formula shows a larger variation by around 1/0.62467. And again, for the Haigis formula, the variation in the a2 constant is lowest followed by the variation in the a1 constant. This is a consequence of the internal formula architecture where the effective lens position is derived from a linear superposition of an intercept a0 and a1/a2 upscaled by ACD/AL. Subfigure c, which displays the CDF for the formula constant derived from a direct formula reversal, shows that the formula constant distributions are very broad and asymmetric with respect to the mean, which is indicated by the vertical dashed black line.

## 4 DISCUSSION

Formula constants and model parameters are highly relevant in the field of cataract surgery to adjust the lens power formula for the best refractive outcome (Aristodemou et al., 2011; Langenbucher et al., 2022; Langenbucher, Szentmáry, Cayless, Müller, et al., 2021; Savini et al., 2020). In general, it is mandatory to report appropriate metrics on the precision and robustness of model parameters (DiCiccio & Efron, 1996; Lopez et al., 2023). In fully disclosed single constant lens formulae, where a direct back calculation of the formula constant for each datapoint is possible, statistical metrics such as the mean, standard deviation, median and confidence interval could give some insight into the precision of the formula constant, even though these metrics do not properly represent the respective metrics from traditional methods with cross‐validation or multiple repetitions of the experiment.

Modern subsampling techniques such as jackknife or bootstrap resampling are widely used in statistics where metrics for precision or robustness of model parameters are required and only one dataset is available (Efron, 1982). Resampling is a way of reusing data to generate hypothetical samples (or resamples) that are representative for the underlying population. Currently, bootstrapping, which involves sampling with replacement (Davison & Hinkley, 1997), is the most popular resampling technique. Bootstrapping has the ability to estimate the sampling distribution of formula constants for all types of formulae, including those with single or multiple constants, and it can be used with both disclosed and black‐box formulae. The main focus here is to evaluate the variance of the formula constants (DiCiccio & Efron, 1996; Lopez et al., 2023). However, bootstrapping is also a perfect tool to estimate the standard deviation, standard error, confidence intervals for normally or non‐normally distributed formula constant data (Davison & Hinkley, 1997). By contrast, jackknife works by sequentially deleting one datapoint in the dataset and computing the formula constants with each of the resulting datasets containing *N* − 1 datapoints (Chavance, 1992). Computation is much simpler than bootstrapping, and the process results in exactly *N* jackknife samples that do not depend on any random sequence generator. Since only one datapoint is left out for each calculation, the distribution of the resulting formula constants is systematically narrower. For this purpose, the distributions are corrected by a scaling factor of $\sqrt{N-1}$, which has already been taken into account in the descriptive data shown in Tables 2 and 3. The main focus of jackknife resampling in our context is to elaborate and reduce the model bias and to evaluate the variance of the formula constants (Liu et al., 1997). However, jackknife is also a suitable tool for estimating the standard deviation, standard error and confidence intervals for formula constant data, and has the great advantage that the mean of all jackknife resampled formula constants fully matches the formula constants derived from the original dataset, which is not necessarily the case with bootstrapping (Davison & Hinkley, 1997; Efron, 1982).

In this paper, we have used jackknife and bootstrap resampling techniques to evaluate the precision or robustness of the formula constants. In contrast to a previous paper where we bootstrapped the formula prediction error PE and added this bootstrapped PE to the formula predicted spherical equivalent refraction (Langenbucher et al., 2023b), in this paper we bootstrapped the entire dataset (with all input parameters) to create a level playing field with jackknife resampling. As shown by Gatinel et al., an overestimation or underestimation in any of the formula constants which directly affect the effective lens position causes a PE of about minus or plus 1.35 dioptres (0.0006·(PIOL^2^ + 2·K12·PIOL)) (Gatinel et al., 2024). Since the Aconst of the SRK/T formula is translated to a shift in the effective lens position of Aconst/0.62467, an overestimation or underestimation in the Aconst value causes a PE of about minus or plus 0.86 dioptres.

The main findings are that both the jackknife and bootstrap resampling techniques are suitable for generating relevant metrics such as the mean, SD, median and CI95 of the formula constants. We could confirm that the mean formula constants from jackknife resampling correspond exactly to the formula constant derived from the original dataset, whereas the mean formula constant from bootstrap resampling shows a slight offset, at least when using a reasonable value for *N*_*B*_. In Tables 2 and 3 the formula constants derived from the original dataset are listed together with the metrics for the formula constants derived from jackknife and bootstrap resampling and from formula reversion. For dataset 1 with the aspherical aberration correcting Hoya lens, the standard deviations for the lens constants of the Hoffer‐Q, Holladay 1, simplified Haigis and Olsen formula range between 0.009 and 0.011 for both resampling techniques and for the SRK/T formula, the range is about 0.018. For the lens formulae with constant triplets, the situation is completely different and depends on the internal architecture of the formula: With the Haigis formula where the effective lens position is given by a linear superposition of an intercept a0 and two regression terms a1·ACD and a2·AL, any variation in a1 or a2 directly affects the intercept a0. Since a1 is scaled by the ACD (with a mean value of 3.19 mm in dataset 1) and a2 is scaled by the AL (with a mean value of 24.10 mm in dataset 1), the SD in a0 (about 0.036) is consequently larger compared with the SD in a1 (about0.006) or a2 (about 0.002) or to the respective SD of a0 in the simplified Haigis formula or the Hoffer‐Q, Holladay 1 or Olsen formulae. This does not, however, affect the accuracy of the predicted effective lens position, as all constants in the constant triplet act together to define the effective lens position and subsequently the formula predicted refraction. The situation for the Castrop formula is rather different as only C scales with the LT (with a mean value of 4.62 mm in dataset 1) and H affects the effective lens position, whereas R acts as an offset value for the refraction. This explains why the SD in C, which is upscaled with LT, is systematically smaller compared with the SD in H. For dataset 2 with the spherical Alcon lens, the standard deviations for the lens constants of the SRK/T, Hoffer‐Q, Holladay 1, simplified Haigis and Olsen formula with both resampling techniques are slightly larger, but they are still in the same range compared with dataset 1. For the lens formulae with constant triplets, SD for the Haigis formula constants a0/a1/a2 is about 0.037/0.006/0.002 and for the Castrop formula SD for C/H/R is about 0.004/0.023/0.013 with jackknife resampling or 0.003/0.015/0.010 with bootstrap resampling. However, the most important finding is that formula constants derived from direct formula reversal perform poorly for all formulae. For dataset 1 with the Hoya lens, the SD for the SRK/T formula is 0.57 and for the Hoffer‐Q/Holladay 1/simplified Haigis/Olsen formulae it is 0.38/0.38/0.32/0.32. Looking at the distributions shown in the CDF plots in Figure 1c centred to their distribution mean value, it can be clearly seen that the distributions of the formula constants are skewed. Also, comparing the formula constant mean or median values listed in Table 2 to the formula constant derived from the entire dataset with nonlinear optimisation shows a systematic offset. As an example, with the Hoffer‐Q formula, the mean/median value reads 5.810/5.755 compared with 5.736. Similar findings are obtained with dataset 2 with the Alcon lens: The SD for the SRK/T formula is 0.57, and for the Hoffer‐Q/Holladay 1/simplified Haigis/Olsen formula, it is 0.38/0.38/0.32/0.32. The distributions shown in the CDF plots in Figure 2c centred to their distribution mean value again show some skewness, and comparison of the formula constant mean or median values listed in Table 3 to the formula constant derived from the entire dataset with nonlinear optimisation again reveals a systematic offset. As an example, with the Hoffer‐Q formula, the mean/median value reads 5.479/5.431 compared with 5.409.

However, there are some limitations in the present study: Firstly, our analysis was restricted to two clinical datasets containing *N* = 888 and *N* = 821 datapoints. Our mathematical strategy for evaluating the precision or robustness of formula constants should be applied to more clinical datasets, preferably from various centres for validation. Secondly, we used a nonlinear iterative optimisation strategy to optimise the formula constants for the entire datasets and for all jackknife and bootstrap samples. This optimisation is well‐established and minimises the root‐mean‐squared PE. Using other optimisation metrics may result in somewhat different findings (Langenbucher et al., 2022; Langenbucher, Szentmáry, Cayless, Müller, et al., 2021). And thirdly, in our study, we restricted the resampling to *N*_*B*_ = 1000 bootstrap samples, as we had to run a nonlinear iterative optimisation cycle for each bootstrap and jackknife sample for both datasets and each formula. We argue that with a much larger number of bootstraps, the mean formula constants derived from bootstrap samples might be even closer to the respective formula constants derived from the entire dataset.

In conclusion, this study describes two resampling‐based strategies to estimate the precision or robustness of formula constants in terms of standard deviation and confidence intervals for single and multiple constant disclosed or black‐box formulae. The jackknife resampling is computationally simpler than the bootstrap resampling, and with our clinical datasets, both techniques yielded similar values for the standard deviations and confidence intervals of the formula constants. Optimising formula constants for disclosed single constant formulae using formula reversion cannot be recommended, as this can result in large variations in the constants and asymmetric distributions, making statistical metrics such as the arithmetic mean unreliable.
